# Factors related to functional prognosis in elderly patients after accidental hip fractures: a prospective cohort study

**DOI:** 10.1186/1471-2318-14-124

**Published:** 2014-11-26

**Authors:** Itziar Vergara, Kalliopi Vrotsou, Miren Orive, Nerea Gonzalez, Susana Garcia, Jose M Quintana

**Affiliations:** Primary Care Research Unit Gipuzkoa, Osakidetza, Paseo Dr Beguiristain s/n, San Sebastian-Donostia, 20014 Spain; Galdakao-Usansolo Hospital Research Unit, Galdakao, Bizkaia Spain; Health Services Research on Chronic Patients Network (REDISSEC), San Sebastian-Donostia, Spain; Biodonostia Health Research Institute, San Sebastian-Donostia, Spain

**Keywords:** Hip fractures, Elderly, Cohort study

## Abstract

**Background:**

A restriction in functional capacity occurs in all hip fractures and a variety of factors have been shown to influence patient functional outcome. This study sought to provide new and comprehensive insights into the role of factors influencing functional recovery six months after an accidental hip fracture.

**Methods:**

A prospective cohort study was conducted of patients aged 65 years or more who attended the Emergency Room (ER) for a hip fracture due to a fall. The following were studied as independent factors: socio-demographic data (age, sex, instruction level, living condition, received help), comorbidities, characteristics of the fracture, treatment performed, destination at discharge, health-related quality of life (12-Item Short Form Health Survey) and hip function (Short Western Ontario and McMaster Universities Osteoarthritis Index). As main outcome functional status was measured (Barthel Index and Lawton Instrumental Activities of Daily Living Scale). Data were collected during the first week after fracture occurrence and after 6 months of follow-up. Patients were considered to have deteriorated if there was worsening in their functional status as measured by Barthel Index and Lawton IADL scores. Factors associated with the outcome were studied via logistic regression analysis.

**Results:**

Six months after the fall, deterioration in function was notable, with mean reductions of 23.7 (25.2) and 1.6 (2.2) in the Barthel Index and Lawton IADL Scale scores respectively. Patients whose status deteriorated were older, had a higher degree of comorbidity and were less educated than those who remained stable or improved. The multivariate model assessing the simultaneous impact of various factors on the functional prognosis showed that older patients, living with a relative or receiving some kind of social support and those with limited hip function before the fall had the highest odds of having losses in function.

**Conclusion:**

In our setting, the functional prognosis of patients is determined by clinical and social factors, already present before the occurrence of the fracture. This could make it necessary to perform comprehensive assessments for patients with hip fractures in order to identify those with a poor functional prognosis to tackle their specific needs and improve their recovery.

## Background

Episodes of fall and consequent injury among community-dwelling elderly populations are a major issue in developed countries, from a clinical and public health perspective [1]. The prevalence of falls in elderly people has been found to be between 14 and 32% according to various epidemiological studies [2–4]. These rates have remained steady in our setting over the last fifteen years [5].

Falls have severe consequences in elderly individuals [6], from a physical [6, 7] as well as a psychological perspective [8]. Among community-dwelling elderly people, the prevalence of fractures after falls varies between 7.8 and 16.5% [4, 5]. The most frequent fractures from a fall are hip and Colles fractures [3], and the most frequent severe complication of a fall event is the occurrence of a hip fracture.

Hip fracture is a significant cause of morbidity and mortality worldwide. It is estimated that hip fractures are responsible for 1.75 million disability adjusted life-years lost, representing 0.1% of the global burden of disease worldwide and 1.4% of the burden amongst women from the established market economies [9]. Having a hip fracture is considered one of the most fatal fractures for elderly people, resulting in impaired function, and increased morbidity and mortality. Functional capacity, specifically related to activities of daily living, is restricted in all hip fractures, and according to published data elderly individuals with hip fracture do not reach their pre-fracture levels of functioning one year post-fracture in 29 to 50% of cases [10, 11]. Hip fractures are associated with a pronounced decline in physical functioning at 2 years, independent of the effects of increasing age, pre-existing medical conditions and disabilities [12].

A variety of factors have been shown to influence patient outcome after an accidental hip fracture; these include: age, pre-fracture functioning and health status, fracture type, associated pain, anemia, dementia, muscle strength, and early mobility level [13, 14]. Thus, the outcome of patients with hip fracture is considered multi-factorial, not being possible to explain it with just one or two single factors [15].

This paper presents new data on the role of factors influencing functional prognosis after accidental hip fracture in a comprehensive manner, including traditionally studied individual-based factors such as sex, age, previous level of functioning and health status, but also, other factors related to the medical care provided and the socioeconomic sphere.

## Methods

The study was based on data pertaining to a prospective cohort study of six months of follow up, carried out with patients aged 65 years or more who attended the Emergency Room (ER) for a hip fracture due to a fall. Six public teaching hospitals of the Basque Health Service (Osakidetza) took part in this study. Osakidetza provides near-universal public health coverage for 2 million people in the Basque Country, an autonomous region in northern Spain. All participating hospitals have similar populations and offer similar levels of technical performance. All patients were informed about the study, and gave written informed consent before inclusion. Ethics Committee of Hospital Galdakao-Usansolo approved the study.

Patients with physical or psychological impairments that prevented them from properly completing the questionnaires were excluded from the study, as were any cases in which syncope [16] was identified as the main cause of the fall or a pathologic fracture was suspected. Those not interested in taking part in the study were also excluded. Patients who completed less than 50% of the questionnaires or those who decided not to answer them were considered losses to follow-up.

Information was collected at two time points: at baseline, at the time the patient was attended at the ER due to the fall, and 6 months after the fall. Baseline information was obtained from medical records, from both the ER database and the hospital medical record, and through personal interviews. These interviews took place always during the first week after the fall. Baseline information included the following: from the ER medical record, socio-demographic data, characteristics of the fracture, diagnostic tests performed, proposed treatment, and destination at discharge; and from the hospital medical records, comorbidity (Charlson Index), fracture severity (Müller AO/OTA Classification), treatment of the fracture (reduction, immobilization, surgery), hospital admission (length of stay, complications), and destination at discharge (home, residence, long term hospital), or date of death. During the personal interview the following were assessed: characteristics of patients’ social support network, level of education, income, self-reported health-related quality of life and functionality, both before the fall (retrospectively) and at the moment of the interview,.

Patients were assessed 6 months after the fall by reviewing their clinical records and by the completion of questionnaires containing the same instruments as those used in the baseline examination. These questionnaires were sent to all the participants by mail, and in order to minimize losses, participants were carefully followed-up. Those who did not return the materials were sent a reminder letter at 21 days and again at 35 days, if needed. After that, participants were telephoned to increase the response rate and, as required, to adapt the interview procedure to the preferences of the participants, conducting the questionnaires over the telephone for those with visual impairments, for example. In the event that a telephone interview was needed, it was performed by the same trained interviewers.

The most important instruments used are briefly described in the following paragraphs. Functionality was assessed with the Barthel Index [17, 18], in order to explore patients’ ability to perform basic activities of daily living (BADL), and the Lawton Scale [19, 20] for instrumental daily living activities (IADL). These two constitute the main outcomes studied. The Barthel Index consists of 10 items that measure a person’s daily functioning, specifically activities of daily living and mobility. The items cover feeding, moving from wheelchair to bed and back, grooming, transferring to and from a toilet, bathing, walking on a level surface, going up and down stairs, dressing, and bowel and bladder control. The assessment can be used to determine a baseline level of functioning and to monitor changes in ability to perform activities of daily living over time. The scores for each of the items are summed to give a total score. Possible scores range from 0 to 100, with lower scores indicating more severe disability. Independence is taken to mean that the person needs no assistance with any part of the task. The Lawton Scale was developed by Lawton and Brody to assess complex activities of daily livings for older adults living in the community. Composed of 8 items, it assesses a person’s ability to perform tasks such as using a telephone, doing laundry, and handling finances. Responses to each of the eight items in the scale are coded as 0 (unable or partially able) or 1 (able), and the eight responses are summed. Accordingly, the summary score ranges from 0 (low function, dependent) to 8 (high function, independent).

A specific questionnaire was used to measure hip function and symptoms, namely, the short version of the Western Ontario and McMaster Universities Osteoarthritis Index (WOMAC) [21, 22]. The WOMAC is a disease-specific, self-administered questionnaire developed to assess hip symptomatology and function in patients with hip or knee osteoarthritis and it has also been applied in patients with hip fracture [23]. The short form (WOMAC-SF) used in this study has 11 items grouped into two dimensions: pain (3 items) and function [LCF] (8 items). The final scores were determined by adding the aggregate scores for pain and function separately, standardizing them to range from 0 to 100, with 0 representing the best health status possible and 100 the worst.

In addition, Health-related quality of life (HRQoL) was evaluated, with the 12-Item Short Form Health Survey (SF-12) [24, 25]. The SF-12 Health Survey is a generic instrument for measuring HRQoL. The SF-12 contains 12 items from the SF-36 Health Survey [26], to reproduce the physical component summary score (PCS) and the mental component summary (MCS) scores. The PCS and MCS scores are calculated from the responses to 12 questions and range from 0 to 100, where zero indicates the poorest level of health measured by the scales and 100 the best level.

Validated Spanish language versions of these aforementioned questionnaires were used.

### Statistical analysis

Categorical data are presented as frequencies with percentages (%) and continuous data as means with standard deviations (SDs). Associations between categorical variables were assessed with the chi-square test. The two-sample t-test and Mann–Whitney test were implemented for two group comparisons of continuous variables. P-values <0.05 were considered statistically significant.

In order to perform the necessary analysis, patients were categorized into two groups according to the change in their functional status, defined as the difference between the scores on Barthel Index and Lawton IADL scales at baseline and 6 months after the fracture. Regarding the Barthel Index, patients’ condition was considered to have deteriorated if they obtained post-fall scores of <90 points or their score decreased by more than 10%, given that 90 points is defined as a threshold for moderate dependency and that a 10% decrease may imply in some cases, a change in the level of independence [27]. With the Lawton IADL Scale, post-fall values of <5 points or a decrease of 2 points was considered to indicate deterioration, taking into account the responsiveness of this test [20]. Analysis was performed separately with the Barthel Index and Lawton IADL Scale results, and also from the perspective of global functional decline, defining a combined variable that considered patients’ status to have deteriorated if either their Barthel Index or Lawton IADL Scale scores dropped by aforementioned amounts.

Patient reported outcome (PRO) measures were compared in the two deterioration groups as a function of baseline values, as well as of pre- to post-fall differences. In order that negative values indicated deterioration, differences were calculated as post-pre values for most PRO measures considered. The exception was WOMAC, for which differences were calculated as pre-post values, for the same reason.

Univariate and multivariate logistic regression models were fitted. The multivariate regression model was constructed with backward selection procedure, initially considering all variables with p-values ≤0.10. Regression results are presented as odds ratios (ORs) and 95% confidence intervals (CIs). Estimations related to SF-12 and short-WOMAC correspond to 10-unit score differences, as the respective score values were transformed accordingly prior to model fitting. The performance of the model was assessed using deviance residuals, the Hosmer-Lemeshow test and Area Under the Curve (AUC) analysis. All analyses were performed with SAS software version 9.3.

## Results

In total, 857 patients were initially included in the study, all having attended the ER services of one of the six participating hospitals for a hip fracture following an accidental fall. From these, 638 fulfilled the inclusion criteria and were actually followed up for six months.

**Figure 1:**
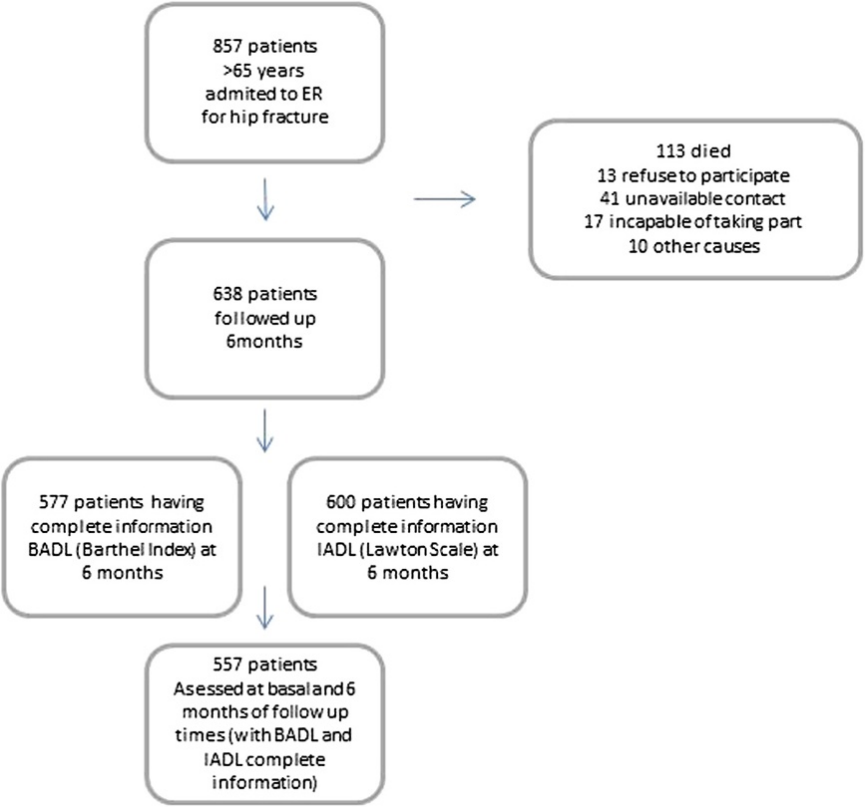
**Flow chart of the recruitment and follow-up process.**

A flow chart of the recruitment and follow-up process is included in Figure 1. Non-responders were found to be significantly older (p = 0.0001), more likely to be male (p < 0.0001), and more likely to have congestive heart failure, dementia or malignant conditions (p < 0.05) than responders. Of the 557 patients who were assessed, 84% were women and the mean age was 83.2 (SD 7.2) years, 48% being 85 or over and only 11% being younger than 75 years old at the time of the study. Regarding the degree of basal comorbidity, only 7% of patients ranked 0 on Charlson Index whereas 78% ranked 2 or more points. Most patients (93%) scored 1 or more on the Charlson Index and were considered to have comorbidity. The most prevalent conditions were COPD, present in 22% of studied patients, type II diabetes and osteoporosis (diagnosed in 20% of patients), and cardiovascular disease (13%). Over half were able to read and write but had no qualifications, and just 2% had a university degree. Almost one third of the sample (29%) was receiving some kind of support from public social services. In addition, the majority (67%) lived with their spouse or a relative before the accidental fall. Regarding pre-fall functional status, the mean scores were 87 (SD 21) on the Barthel index and 4.8 (SD2.9) on Lawton’s IADL Scale. The most frequent fracture types were intertrochanteric and sub-capital fractures. From the ER, patients were transferred to a traumatology ward at the same hospital (92%), or discharged to their homes (3.6%), or to another hospital ward.

**Figure 2:**
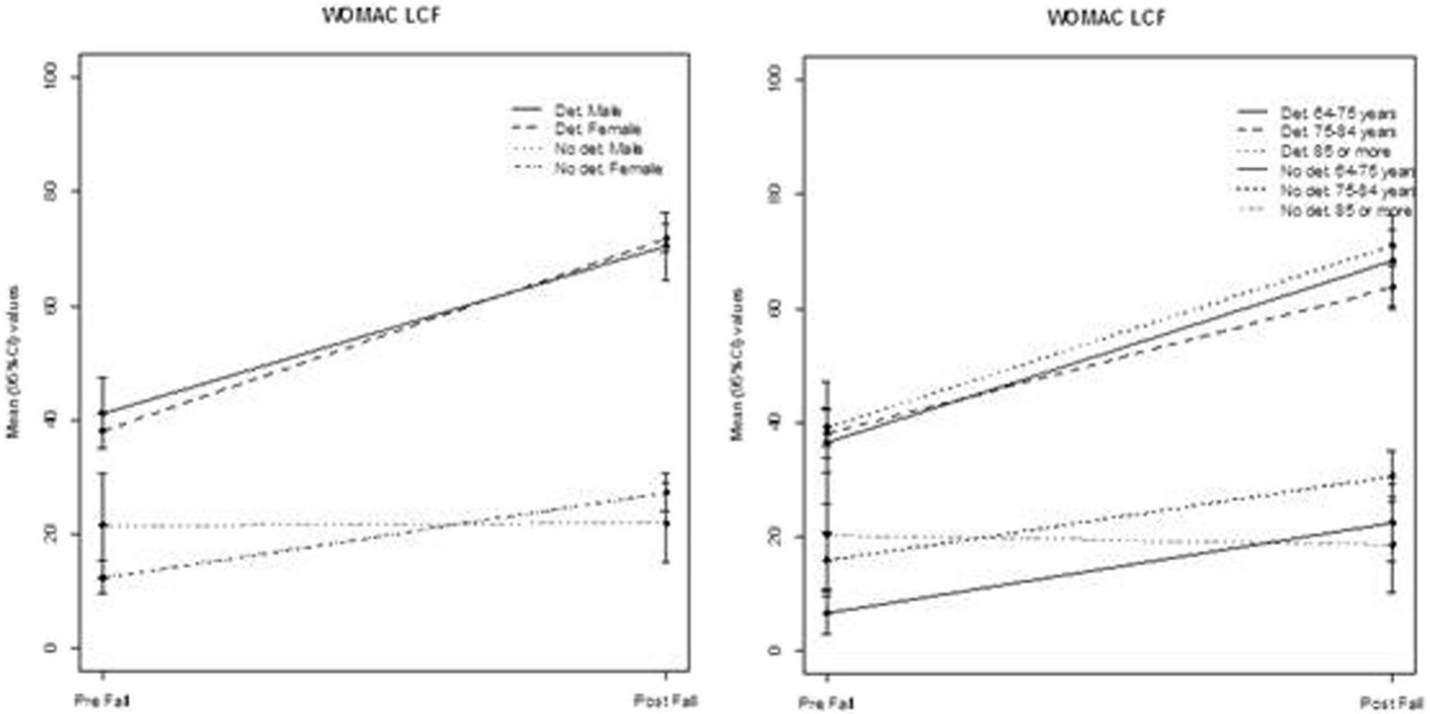
**Hip function and health related quality of life change over six months: Hip function and health related quality of life change over six months of follow up by sex and age group for patients whose functional status had and had not deteriorated.**

Six months after the fall, deterioration in function was notable, with both Barthel Index and Lawton IADL Scale values showing mean reductions of 23.7 (25.2) and 1.6 (2.2) respectively. Considering final functional status with respect to BADL and IADL, 397 and 418 of the 557 subjects deteriorated respectively. Patients showing deterioration in any of these functional capacities were compared with patients whose status had not deteriorated. Their baseline characteristics are presented in Table 1. For both types of functioning, patients whose status had deteriorated were older, were less-well educated, more likely to have comorbidity and polipharmacy, be living with a relative and be receiving some kind of social support. There were also differences when examining the SF-12 and WOMAC scores (Table 2). Calculated differences (pre-post) were more pronounced in WOMAC domains, with patients showing deterioration experiencing greater functional and pain limitations. Women reported more pronounced losses in hip function among both those who had and had not deteriorated, though these differences were not statistically significant. The observed changes in these scores 6 months after the fall are presented in Figure 2, by sex and age group.

**Table 1:** **Baseline characteristics of the total sample as a function of subsequent deterioration in ability to perform BADL and IADL**

Variable	BADL performance			IADL performance		
	Deteriorated (n = 397)	Not deteriorated (n = 160)	p-value	Deteriorated (n = 418)	Not deteriorated (n = 139)	p-value
**Age; mean(SD)**	84.7(6.8)	79.5 (6.6)	<0.0001	84.8 (6.6)	78.3 (6.7)	0.004
**Sex**						
Female	332 (84)	138 (86)	0.440	348 (83)	122 (88)	0.204
**Comorbidities**						
Yes	364 (95)	139 (88)	0.008	383(94)	120 (88)	0.009
**Chalson Index**						
0	21 (6)	19 (12)	0.0003	23 (6)	17 (12)	0.0002
1	48 (12)	34 (22)		51 (12)	31 (23)	
≥2	316 (82)	105 (66)		332 (82)	89 (65)	
**Current medication use**						
None	19 (5)	18 (11)	0.0001	21 (5)	18 (13)	0.0004
1-3 medications	194 (49)	98 (62)		212 (51)	80 (58)	
≥ 4 medications	178 (45)	43 (27)		181 (44)	40 (29)	
**Level of education**						
Illiterate	15 (4)	1 (1)	0.004	14 (3)	2 (1)	0.001
Able to read & write	211 (54)	64 (40)		225 (55)	50 (36)	
Primary education	140 (36)	80 (50)		147 (36)	73 (53)	
Secondary education	17 (4)	10 (6)		19 (4)	8 (6)	
University qualifications	8 (2)	4 (3)		7 (2)	5 (4)	
**Pre-fall living status**						
Alone	12 (3)	13 (8)		13 (3)	12 (9)	0.020
Alone, receiving social support	118 (30)	42 (26)		125 (30)	35 (25)	
With a relative	264 (67)	105 (66)	0.003	227 (67)	92 (66)	
**Pre-fall institutional help**						
Yes	103 (27)	12 (8)	<0.0001	101 (25)	14 (10)	0.0002

Values in cells are frequency (percentage) unless otherwise stated. For variables with missing data frequencies do not add up to N. SD: standard deviation. Patients’ ability to perform basic activities of daily living (BADL) was assessed using the Barthel Index; their functional status was considered to have deteriorated if they obtained post-fall scores of <90 points or a pre-post score decrease of more than 10%. Patients’ ability to perform instrumental activities of daily living (IADL) was assessed using the Lawton Scale; their functional status was considered to have deteriorated if they obtained post-fall scores of <5 points or a pre-post score decrease of 2 points. For binary variables only one category is presented. The p-value columns refer to comparisons between patients whose functional status had and had not deteriorated considering the results of each questionnaire separately.

**Table 2:** **Comparison of baseline values and 6 months post-fall changes in four patient reported outcomes in patients whose ability to perform BADL and IADL had and had not deteriorated**

	BADL performance			IADL performance		
PRO measure	Deteriorated (n = 397)	Not deteriorated (n = 160)	***p-value***	Deteriorated (n = 418)	Not deteriorated (n = 139)	***p-value***
**WOMAC: LCF**						
Baseline	41.4 (27.5)	14.8 (18.6)	*<0.0001*	39.4 (28.1)	16.9 (19.7)	*<0.0001*
Pre-Post	-30.2 (25.6)	-16.5 (23.2)	*<0.0001*	-30.7 (25.0)	-13.0 (23.0)	*<0.0001*
**WOMAC: Pain**						
Baseline	14.5 (21.8)	7.9 (15.5)	*<0.0001*	14.2 (21.6)	7.7 (15.5)	*0.0001*
Pre-Post	-13.5 (33.7)	-7.4 (22.2)	*0.015*	-13.0 (33.2)	-7.9 (22.5)	*0.047*
**SF-12 PCS**						
Baseline	37.3 (10.3)	46.2 (9.6)	*<0.0001*	38.1 (10.6)	45.0 (10.0)	*<0.0001*
Post-Pre	-9.2 (11.6)	-9.2 (10.6)	*0.996*	-9.8 (11.5)	-7.8 (10.5)	*0.101*
**SF-12 MCS**						
Baseline	49.8 (11.8)	52.9 (9.7)	*0.002*	49.7 (11.8)	53.7 (9.3)	*0.0003*
Post-Pre	-4.8 (14.6)	-1.3 (12.2)	*0.010*	-4.3 (14.4)	-2.1 (12.8)	*0.148*
**Barthel**						
Baseline	81.9 (23.5)	98.1 (5.1)	*<0.0001*	82.6 (23.2)	98.3 (4.5)	*<0.0001*
Post-Pre	-33.1 (-23.9)	-0.1 (4.8)	*-*	-30.3 (25.3)	-3.7 (9.4)	*<0.0001*
**Lawton**						
Baseline	4.0 (2.9)	6.9 (1.8)	*<0.0001*	4.0 (2.9)	7.1 (1.4)	*<0.0001*
Post-Pre	-2.0 (2.3)	-0.6 (1.6)	*<0.0001*	-2.2 (2.1)	0.1 (1.1)	*-*

Values are Mean (SD). Changes have been calculated as post minus pre values (Post-Pre), except in the case of WOMAC for which differences have been calculated as Pre-Post. In all cases, negative differences indicate deterioration in patient functional status. Patients’ ability to perform basic activities of daily living (BADL) was assessed using the Barthel Index; their functional status was considered to have deteriorated if they obtained post-fall scores of <90 points or a pre-post score decrease of more than 10%. Patients’ ability to perform instrumental activities of daily living (IADL) was assessed using the Lawton Scale; their functional status was considered to have deteriorated if they obtained post fall scores of <5 points or a pre-post score decrease of 2 points. Changes in Barthel and Lawton scores were not compared between the groups, as these values were used for establishing the groups. LCF (WOMAC physical function domain); SF-12 PCS (SF-12 physical domain); SF-12 MCS (SF-12 mental domain).

No differences were observed regarding the treatment received for the fracture, destination at discharge, indications for clinical follow-up (primary care physician, traumatologist, rehabilitation service), or even regarding satisfaction with the care received (data not shown). Patients whose status had not deteriorated were more likely to return to their own homes (72%) while those who showed deterioration were more likely to repeatedly attend ER services (21% vs 13%) and to be admitted to a hospital for causes unrelated to the fracture (15% vs 6%).

Multivariate models assessing the simultaneous impact of various factors on functional progression are presented (Table 3) for BADL performance, for IADL performance and finally, considering a combined variable representing overall functional deterioration. Variables included systematically for all the three models were age, HRQoL (mental and physical domains of the SF-12) and hip function and pain prior to the fracture. The BADL model also included the presence of cerebrovascular disease and IADL model living conditions prior to the fracture. According to these models, older patients have higher odds of deterioration after an accidental fall. Higher scores of baseline HRQoL, both physical and mental, are associated with a lower probability of having a reduced ability to perform BADL, IADL or both. Poorer hip function prior to the fracture is associated with poorer functional recovery. Lastly, having previously been living with a relative or living alone but receiving some kind of social support, are also associated with functional deterioration.

**Table 3:** **Multivariate logistic regression models for BADL, IADL and global function assessment at 6 months**

	BADL model		IADL model		Global model	
Variable	OR (95% CI)	p-value	OR (95% CI)	p-value	OR (95% CI)	p-value
Age	1.10 (1.07, 1.14)	<0.0001	1.16 (1.12, 1.20)	<0.0001	1.15(1.11, 1.20)	<0.0001
**Sex**						
Male	Ref.		Ref.		Ref.	
Female	1.09 (0.57, 2.06)	0.801	0.87 (0.44, 1.70)	0.675	1.24 (0.60, 2.59)	0.445
**Cerebrovascular disease**						
No	Ref.	-	-	-	-	-
Yes	3.04 (1.11, 8.34 )	0.031	-	-	-	-
**Baseline HRQoL**						
SF-12 PCS	0.69 (0.52, 0.92)	0.010	-	-	-	-
SF-12 MCS	0.75 (0.60, 0.94)	0.012	0.66 (0.52, 0.84)	0.001	0.70 (0.54, 0.92)	0.011
**LCF of womac**	1.36 (1.20, 1.55)	<0.0001	1.36 (1.23, 1.51)	<0.0001	1.47 (1.30, 1.67)	<0.0001
**Living status before the fall**						
Alone	-	-	Ref.	-	Ref.	-
Alone, receiving social support	-	-	2.44 (0.87, 6.86)	0.091	3.79 (1.28, 11.21)	0.023
With a relative	-	-	3.29 (1.23, 8.83 )	0.018	3.92 (1.42, 10.79)	0.013
Goodness–of-fit statistics						
Hosmer-Lemeshow	p = 0.702		p = 0.869		p = 0.310	
R square/adjusted R square	0.275 / 0.394		0.257 / 0.380		0.244 / 0.389	
AUC	0.835		0.829		0.847	

OR: Odds Ratio; 95% CI: 95% Confidence Intervals. BADL model: multivariate model considering status at 6 months (deteriorated or not) based on ability to perform basic activities of daily living (BADL) as assessed using the Barthel Index; patients’ functional status was considered to have deteriorated if they obtained post-fall scores of <90 points or a pre-post score decrease of more than 10%. IADL model: multivariate model considering status at 6 months based on ability to perform instrumental activities of daily living (IADL) as assessed using Lawton Scale; patient functional status was considered to have deteriorated if they obtained post fall scores of <5 points or a pre-post score decrease of 2 points. Global model: multivariate model considering status at 6 months based jointly on BADL and IADL assessments. Estimations presented: for age refer to 1-unit increases; and for baseline health-related quality of life (HRQoL) refer to 10-unit increases in the respective score scales. LCF (WOMAC physical function domain); SF-12 PCS (SF-12 physical domain); SF-12 MCS (SF-12 mental domain); AUC (area under the curve).

The three derived models presented adjusted R^2^ > 23%, and AUC > 0.80.

## Discussion and conclusions

In our setting, patients with a hip fracture after an accidental fall are generally women, very old (over 85), with several chronic conditions and comorbidity, polipharmacy and a borderline functional status (almost dependent). From the social perspective, they tend to have a lower level of education and be living with relatives.

The functional performance of these patients is still severely impaired 6 months after their fall. Severe deterioration in all studied aspects of functioning is observed, specifically in the ability to perform basic and instrumental activities of daily living and also in specific hip function, both in terms of functional limitations and pain. HRQoL evolved in a similar way in both the physical and mental domains and the changes are much more pronounced in patients with overall functional capacities that were lower 6 months after the fall (compared to before the fall).

Notably, the functional recovery is not related to the circumstances or characteristics of the fracture, considering the most frequent types assessed in this study, or with the treatment received, either at ER or after discharge. Rather, the functional outcome of these events is related to individual characteristics of the patients, specifically their previous health and functional status and their social and living circumstances. Specifically, patients whose status worsened were significantly older, presented higher degree of comorbidity, and were less educated than those who did not. Further, regarding living conditions, patients who deteriorated were more likely to have been living with a relative or receiving support from social services.

Several population-based prospective cohort studies have shown functional prognosis to be negatively associated with cognitive impairment, advanced age, more comorbidities, hip pain and function, poor self-rated health, and depression symptoms [13, 28, 14]. One study based on 338 community-dwelling elderly patients found that those living alone were at a higher risk of delay or failure in recovering ability to perform BADL [14]. Regarding the characteristics of the fracture, association was not found between the type of fracture and the functional recovery, even though type fracture is clearly associated with mortality [29–31]. With regard to the type of treatment, differences were not found in six months functional recovery between those receiving internal fixation of prosthetic replacement. Similar results have been described even though differences were observed in the short term functional performance during hospitalization [32].

Our results mostly support these previous findings, and provide additional insight into the complexity of factors determining functional prognosis. The models constructed based on deterioration in ability to perform BADL and IADL and also the summary functional outcome are very similar and allow us to establish the profile of patients with a high likelihood of having a poor functional prognosis. This profile is strongly related to the baseline functioning and living conditions, where no factors related to the severity or the characteristics of the fracture show any association with the functional prognosis of aged patients with fractures due to accidental falls. The relevance of their living conditions constitutes the main difference between our findings and those of previous studies. In our case, living alone was associated with the best functional prognosis. This difference is probably due to the baseline characteristic of the samples where other studies have considered community-dwelling older adults but in this one all patients with hip fractures were included. Accordingly, many of our patients were already highly dependent at the baseline and it is likely for disabled patients to be already living with a relative.

The association of disability and frailty with the risk of fractures is well documented [33, 34], and our work provides additional evidence of the role of individual functioning and living conditions in the pathway to an accidental fracture in older adults. Functional assessment of elderly people could provide an effective strategy to identify subjects at risk of sustaining a fracture and of a poor functional prognosis.

It is possible that the high quality of the medical care provided in the health system studied creates a new paradigm, in which the characteristics of the medical care itself have little or no influence on patients’ functional recovery, and individual factors become the most relevant; this would shift the focus of hip fracture treatment towards the individual and social characteristics of patients. Such a phenomenon would require a concurrent shift in the model of care provided, making it necessary to undertake a comprehensive assessment of these patients’ needs and conditions, not only from a medical but also from a social perspective. Integrative models of care implemented early in the natural history of the fracture could be considered.

This study has some limitations. The most important related to the unavoidable fact that baseline health and social status data were collected in a retrospective manner after the fracture. The recall of the previous abilities may be affected by the recognition of current limitations. On the other hand, this information was collected as soon as possible and through standardized instruments that can be expected to help to minimize the recall effect. These instruments were used both, by interviewers and self-completed by the participants which could be considered as an additional limitation, even though, used instruments were suitable for both types of use. An additional limitation is related to the prospective follow-up of subjects and the loss to follow-up of individuals over time, though our response rate (74%) can be considered acceptable [35]. In this case, data regarding the first 6 months of recovery have been considered, in the belief that the main response to treatment and rehabilitation measures would be evident by the end of that period [29]. Finally, it should be underlined that not all the functional deterioration observed in these subjects is necessarily attributable to the fracture and no data are presented for a control group; however, the objective of this study was not to measure the effect of a hip fracture on functional loss, but rather to characterize patients with poor functional prognosis.

Hip fractures constitute a major public health issue, given their prevalence and devastating effect on personal autonomy. It is essential to take a comprehensive approach to social and health care provision for each patient with this type of fracture, as well as adopt effective strategies of disability prevention, to tackle the complex network of determinants of the functional recovery in these individuals.

## References

[1] Sattin RW (1992). Falls among older persons: a public health perspective. Annu Rev Public Health.

[2] Salva A, Bolibar I, Pera G, Arias C (2004). Incidence and consequences of falls among elderly people living in the community. Med Clin (Barc ).

[3] Tinetti ME, Speechley M, Ginter SF (1988). Risk factors for falls among elderly persons living in the community. N Engl J Med.

[4] Varas-Fabra F, Castro ME, de Torres LA P, Fernandez Fernandez MJ, Ruiz MR, Enciso BI (2006). Falls in the elderly in the community: prevalence, consequences, and associated factors. Aten Primaria.

[5] Silva Gama ZA, Gomez CA, Sobral FM (2008). Epidemiology of falls in the elderly in Spain: a systematic review, 2007. Rev Esp Salud Publica.

[6] Campbell AJ, Borrie MJ, Spears GF, Jackson SL, Brown JS, Fitzgerald JL (1990). Circumstances and consequences of falls experienced by a community population 70 years and over during a prospective study. Age Ageing.

[7] Kannus P, Parkkari J, Koskinen S, Niemi S, Palvanen M, Jarvinen M (1999). Fall-induced injuries and deaths among older adults. JAMA.

[8] Arfken CL, Lach HW, Birge SJ, Miller JP (1994). The prevalence and correlates of fear of falling in elderly persons living in the community. Am J Public Health.

[9] Johnell O, Kanis JA (2004). An estimate of the worldwide prevalence, mortality and disability associated with hip fracture. Osteoporos Int.

[10] Bertram M, Norman R, Kemp L, Vos T (2011). Review of the long-term disability associated with hip fractures. Inj Prev.

[11] Flikweert ER, Izaks GJ, Reininga IH, Wendt KW, Stevens M (2013). Evaluation of the effect of a comprehensive multidisciplinary care pathway for hip fractures: design of a controlled study. BMC Musculoskelet Disord.

[12] Norton R, Butler M, Robinson E, Lee-Joe T, Campbell AJ (2000). Declines in physical functioning attributable to hip fracture among older people: a follow-up study of case–control participants. Disabil Rehabil.

[13] Takayama S, Iki M, Kusaka Y, Takagi H, Tamaki S (2001). Factors that influence functional prognosis in elderly patients with hip fracture. Environ Health Prev Med.

[14] Koval KJ, Skovron ML, Aharonoff GB, Zuckerman JD (1998). Predictors of functional recovery after hip fracture in the elderly. Clin Orthop Relat Res.

[15] Kristensen MT (2011). Factors affecting functional prognosis of patients with hip fracture. Eur J Phys Rehabil Med.

[16] Soteriades ES, Evans JC, Larson MG, Chen MH, Chen L, Benjamin EJ, Levy D (2002). Incidence and prognosis of syncope. N Engl J Med.

[17] van der Putten JJ, Hobart JC, Freeman JA, Thompson AJ (1999). Measuring change in disability after inpatient rehabilitation: comparison of the responsiveness of the Barthel index and the Functional Independence Measure. J Neurol Neurosurg Psychiatry.

[18] Baztan JJ, Hornillos M, Gonzalez-Montalvo JI (1993). Geriatric day hospital. Characteristics, performance, and effectiveness. Med Clin (Barc ).

[19] Lawton MP, Brody EM (1969). Assessment of older people: self-maintaining and instrumental activities of daily living. Gerontologist.

[20] Vergara I, Bilbao A, Orive M, Garcia-Gutierrez S, Navarro G, Quintana JM (2012). Validation of the Spanish version of the Lawton IADL Scale for its application in elderly people. Health Qual Life Outcomes.

[21] Bellamy N, Buchanan WW, Goldsmith CH, Campbell J, Stitt LW (1988). Validation study of WOMAC: a health status instrument for measuring clinically important patient relevant outcomes to antirheumatic drug therapy in patients with osteoarthritis of the hip or knee. J Rheumatol.

[22] Bilbao A, Quintana JM, Escobar A, Las HC, Orive M (2011). Validation of a proposed WOMAC short form for patients with hip osteoarthritis. Health Qual Life Outcomes.

[23] Macaulay W, Yoon RS (2008). Fixed-bearing, medial unicondylar knee arthroplasty rapidly improves function and decreases pain: a prospective, single-surgeon outcomes study. J Knee Surg.

[24] Ware J, Kosinski M, Keller SD (1996). A 12-Item Short-Form Health Survey: construction of scales and preliminary tests of reliability and validity. Med Care.

[25] Gandek B, Ware JE, Aaronson NK, Apolone G, Bjorner JB, Brazier JE, Bullinger M, Kaasa S, Leplege A, Prieto L, Sullivan M (1998). Cross-validation of item selection and scoring for the SF-12 Health Survey in nine countries: results from the IQOLA Project. International Quality of Life Assessment. J Clin Epidemiol.

[26] Alonso J, Prieto L, Anto JM (1995). [The Spanish version of the SF-36 Health Survey (the SF-36 health questionnaire): an instrument for measuring clinical results]. Med Clin (Barc ).

[27] Shah S, Vanclay F, Cooper B (1989). Improving the sensitivity of the Barthel Index for stroke rehabilitation. J Clin Epidemiol.

[28] Mossey JM, Mutran E, Knott K, Craik R (1989). Determinants of recovery 12 months after hip fracture: the importance of psychosocial factors. Am J Public Health.

[29] Magaziner J, Fredman L, Hawkes W, Hebel JR, Zimmerman S, Orwig DL, Wehren L (2003). Changes in functional status attributable to hip fracture: a comparison of hip fracture patients to community-dwelling aged. Am J Epidemiol.

[30] Koval KJ, Skovron ML, Aharonoff GB, Meadows SE, Zuckerman JD (1995). Ambulatory ability after hip fracture: a prospective study in geriatric patients. Clin Orthop.

[31] Haentjens P, Autier P, Barette M, Venken K, Vanderschueren D, Boonen S (2007). Survival and functional outcome according to hip fracture type: a one-year prospective cohort study in elderly women with an intertrochanteric or femoral neck fracture. Bone.

[32] Koval KJ, Aharonoff GB, Su ET, Zuckerman JD (1998). Effect of acute inpatient rehabilitation on outcome after fracture of the femoral neck or intertrochanteric fracture. J Bone Joint Surg Am.

[33] Lacas A, Rockwood K (2012). Frailty in primary care: a review of its conceptualization and implications for practice. BMC Med.

[34] Fried LP, Tangen CM, Walston J, Newman AB, Hirsch C, Gottdiener J, Seeman T, Tracy R, Kop WJ, Burke G, McBurnie MA, Cardiovascular Health Study Collaborative Research Group (2001). Frailty in older adults: evidence for a phenotype. J Gerontol A Biol Sci Med Sci.

[35] Cummings SM, Savitz LA, Konrad TR (2001). Reported response rates to mailed physician questionnaires. Health Serv Res.

[CR36] The pre-publication history for this paper can be accessed here: http://www.biomedcentral.com/1471-2318/14/124/prepub

